# Human disease-associated single nucleotide polymorphism changes the orientation of DROSHA on pri-mir-146a

**DOI:** 10.1261/rna.077487.120

**Published:** 2020-12

**Authors:** Cong Truc Le, Thuy Linh Nguyen, Trung Duc Nguyen, Tuan Anh Nguyen

**Affiliations:** Division of Life Science, The Hong Kong University of Science and Technology, Hong Kong, China

**Keywords:** DGCR8, DROSHA, miR-146a, miRNA, single nucleotide polymorphism, rs2910164

## Abstract

The Microprocessor complex of DROSHA and DGCR8 initiates the biosynthesis of microRNAs (miRNAs) by processing primary miRNAs (pri-miRNAs). The Microprocessor can be oriented on pri-miRNAs in opposite directions to generate productive and unproductive cleavages at their basal and apical junctions, respectively. However, only the productive cleavage gives rise to miRNAs. A single nucleotide polymorphism (SNP, rs2910164) in pri-mir-146a is associated with various human diseases. Although this SNP was found to reduce the expression of miRNA, it is still not known if it affects the activity of the Microprocessor directly, and how it functions. In this study, we revealed that the SNP creates an unexpected mGHG motif at the apical junction of pri-mir-146a. This mGHG motif interacts with the double-stranded RNA-binding domain (dsRBD) of DROSHA, switching its orientation on pri-mir-146a from the basal to the apical junction. As a result, the SNP facilitates Microprocessor to cleave SNP-pri-mir-146a at its unproductive sites. Our findings help to elucidate the molecular mechanism that explains how the disease-associated SNP modulates the biogenesis of pri-mir-146a and thereby affects its cellular functions.

## INTRODUCTION

MicroRNAs (miRNAs) are single-stranded RNAs (ssRNAs) that function to silence gene expression. miRNAs interact with the Ago protein to form a core component of an RNA-induced silencing complex (RISC) (Iwasaki and Tomari 2009; Jonas and Izaurralde 2015; Bartel 2018; Gebert and MacRae 2019). The miRNA in RISC often forms 7 bp with the target messenger RNA (mRNA) using its seed sequence between the second to eighth nucleotides from its 5p-end (Jonas and Izaurralde 2015; Bartel 2018). The ability of an miRNA to silence the expression of a gene is mainly determined by its level of expression in the cell; this is primarily controlled by the level of miRNA biogenesis. In human cells, miRNA biogenesis starts in the nucleus with the cleavage of primary miRNA (pri-miRNA) by Microprocessor. This cleavage generates a short stem–loop RNA, called the precursor miRNA (pre-miRNA). Pre-miRNAs usually contain a stem of 22 bp and a loop of varying lengths. In the cytoplasm, DICER removes the loop from the pre-miRNA and this produces a short RNA duplex of ∼22 bp (Ha and Kim 2014; Bartel 2018). Ago then uses one of the strands from this RNA duplex to form an Ago-miRNA complex in the RISC (Kawamata and Tomari 2010; Jonas and Izaurralde 2015; Gebert and MacRae 2019). Therefore, the expression level of the cellular miRNA is governed by each step of its biogenesis, which involves Microprocessor, DICER, and Ago.

**FIGURE 1.**
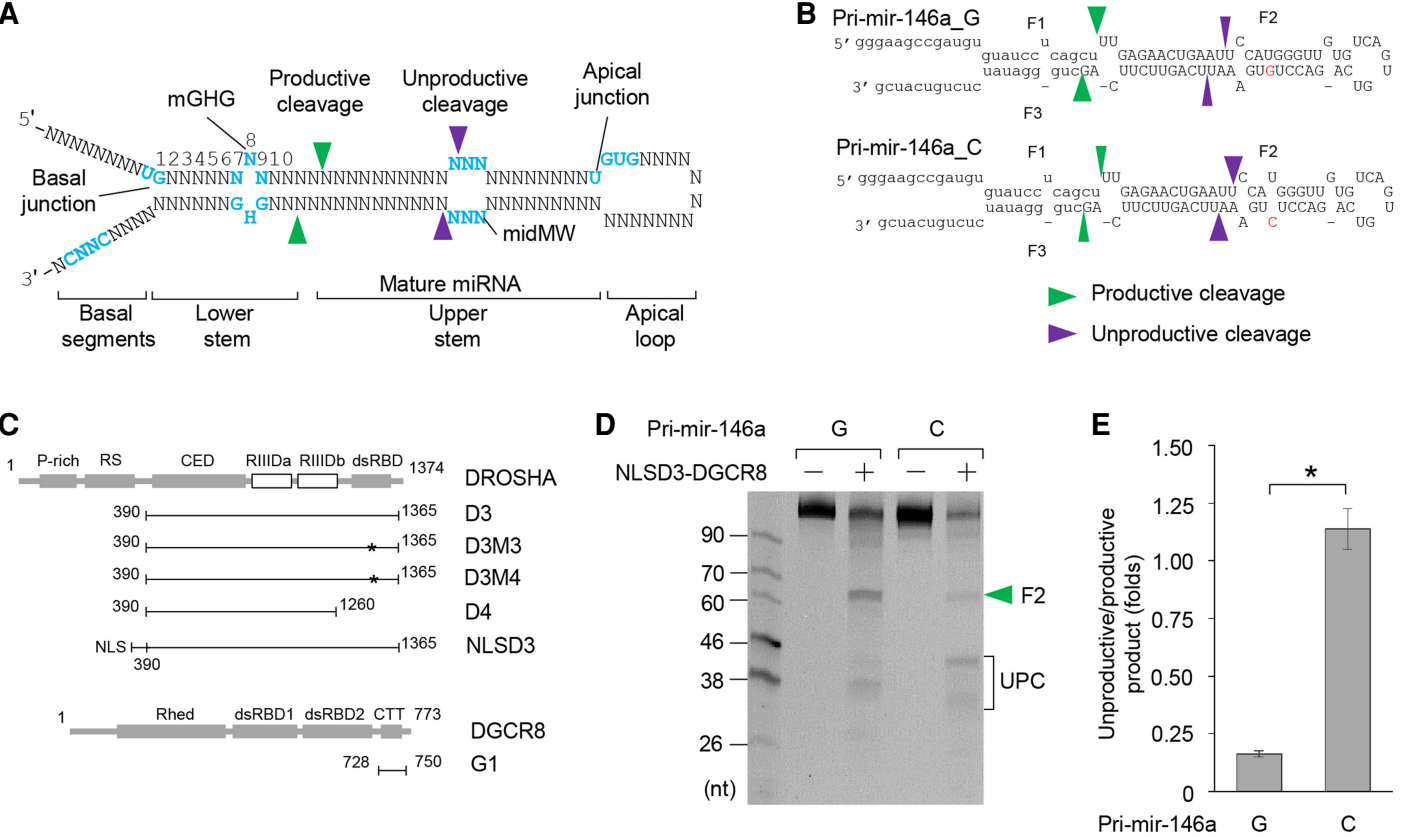
The G/C SNP facilitates the unproductive cleavage of Microprocessor. (*A*) Typical structure of pri-miRNA. (*B*) Structures of pri-mir-146a_G and C. The green and purple arrowheads indicate the productive and unproductive cleavages, respectively. The capital letters represent pre-mir-146a. (*C*) Diagrams of the protein fragments used in this study. The numbers show the positions of the amino acids in each fragment, whereas the asterisks indicate the point mutations. NLS: nuclear localization signal. (*D*) The cleavage of pri-mir-146a_G and C by Microprocessor. The green arrowheads indicate the productive cleavages. UPC indicates the unproductive products. Five pmol of each RNA was incubated with 5 pmol Microprocessor in 10 µL pri-miRNA processing buffer. (*E*) Assessment of the unproductive cleavages by Microprocessor from three independent experiments (an example of which is shown in *D*). The two data sets were significantly different as indicated by the asterisk (*) (two-tailed *t-*test, *P* = 0.031).

Microprocessor consists of a catalytic subunit, DROSHA, and an RNA-binding protein, DGCR8 (Ha and Kim 2014; Bartel 2018; Nguyen et al. 2019), and its major cellular substrates are pri-miRNAs (Fig. 1A). Most pri-miRNAs contain a stem of ∼35 bp and two single-stranded RNA/double-stranded RNA (ssRNA/dsRNA) junctions (Han et al. 2006; Fang and Bartel 2015; Roden et al. 2017). One of the ssRNA/dsRNA junctions (called the basal junction) is formed between the stem and the two basal RNA segments, whereas the other (the apical junction) is formed between the stem and the loop. Microprocessor can cleave pri-miRNAs at either the basal or apical junction since DROSHA recognizes both, and cleaves dsRNA ∼13 bp from each site (Nguyen et al. 2015; Nguyen et al. 2019). However, whereas cleavage at the basal junction generates pre-miRNAs, and thus it is “productive,” cleavage at the apical junction destroys the miRNA sequence, and thus it is “unproductive”.

Multiple RNA elements ensure that DROSHA locates and cleaves pri-miRNAs productively at the basal junction (Nguyen et al. 2019). For example, there is a UG motif, which is highly enriched at the basal junction of many pri-miRNAs, and this recruits DROSHA to the basal junction (Auyeung et al. 2013; Nguyen et al. 2015). An mGHG motif is located in the lower stem, and this interacts with the double-stranded RNA binding domain (dsRBD) of DROSHA, and positions DROSHA at the basal junction (Fang and Bartel 2015; Kwon et al. 2019). Finally, a CNNC motif at the 3p-end basal segment interacts with SRSF3, and this in turn recruits DROSHA to the basal junction (Auyeung et al. 2013; Fernandez et al. 2017; Kim et al. 2018). These three motifs facilitate the localization of DROSHA at the basal junction, and in this way, they increase the likelihood of productive cleavage.

There are also multiple RNA elements, which prevent DROSHA from residing at the apical junction (Nguyen et al. 2019). For example, a UGU motif in the apical loop enhances the interaction between the loop and DGCR8 (Auyeung et al. 2013; Nguyen et al. 2015; Dang et al. 2020). This interaction is further strengthened by hemin, which is associated with DGCR8 (Partin et al. 2017; Nguyen et al. 2018). The presence of DGCR8 at the loop also prevents DROSHA from interacting with the apical junction. In addition, a midMW, which is frequently present in the upper stem of human pri-miRNAs, inhibits the unproductive cleavage by DROSHA (Li et al. 2020). The inhibition of unproductive cleavage by these mechanisms increases the chance of productive cleavages by Microprocessor. Apart from these RNA elements, there are also numerous protein factors, which are reported to control pri-miRNA processing using multiple mechanisms (Ha and Kim 2014; Bartel 2018; Michlewski and Cáceres 2019; Nguyen et al. 2019; Treiber et al. 2019).

miR-146a is synthesized from pri-mir-146a, which is transcribed from the *5q33* locus in chromosome 5. miR-146a is associated with numerous human diseases, including cancer, inflammation, the innate immune response, cardiovascular disease, and both acute and chronic kidney diseases (Paterson and Kriegel 2017). The cellular expression of miR-146a is regulated at the transcriptional level and via single nucleotide polymorphism (SNP). Regarding the former, the transcription factor nuclear factor к-light chain-enhancer of activated B cells (NF-кB) is known to regulate miR-146a expression (Taganov et al. 2006). With regards to the latter, a common G/C SNP of pri-mir-146a, rs2910164, was first identified in samples from papillary thyroid carcinoma (PTC) patients (Jazdzewski et al. 2008). The G allele produces pri-mir-146a (pri-mir-146a_G), which has a U–G wobble base pair in the upper stem (Fig. 1B). In contrast, the C allele generates pri-mir-146a (pri-mir-146a_C), which contains a mismatch (U–C) in the same location as the U–G in pri-mir-146a_G (Fig. 1B). The C allele reduces the expression of miR-146a in human cells (Jazdzewski et al. 2008; Alipoor et al. 2018; Papathanasiou et al. 2020). Furthermore, this type of G/C SNP has been found in many human diseases, including hepatocellular carcinoma, gastric cancer, breast cancer, thyroid carcinogenesis, rheumatoid arthritis, coronary artery diseases, ankylosing spondylitis, colorectal cancer, and cervical cancer (Xu et al. 2008; Kogo et al. 2011; Lian et al. 2012; Wei et al. 2013; Li et al. 2014; Bao et al. 2015; Xu et al. 2015; Bogunia-Kubik et al. 2016; Iguchi et al. 2016; Hu et al. 2018). This association between the G/C SNP and human diseases indicates its essential roles in various clinical and cellular functions. Previously, the G/C SNP was shown to reduce pri-mir-146a processing using in vitro pri-miRNA processing assays and nuclear extract from Hela cells (Jazdzewski et al. 2008). However, whether the G/C SNP directly inhibits the pri-miRNA processing activity of Microprocessor and how it achieves this, remains unknown. In this study, we used in vitro pri-miRNA processing assays to investigate the molecular mechanism of the G/C SNP in pri-miRNA processing. Using synthetic pri-mir-146a and different purified Microprocessor complexes, we demonstrated that the G/C SNP creates an unexpected mGHG motif at the apical junction and consequently recruits DROSHA to this junction. This recruitment is dependent on the dsRBD of DROSHA. As a result, the G/C SNP increases the unproductive cleavage capacity of Microprocessor, and reduced the production of pre-mir-146a and miR-146a. Together, our findings reveal how the G/C SNP in pri-mir-146a controls miR-146a biogenesis.

## RESULTS

### The G/C SNP facilitates unproductive cleavage by Microprocessor

We examined the cleavage activity of the Microprocessor complex (NLSD3–DGCR8) on pri-mir-146a_G and C (Fig. 1B). The NLSD3–DGCR8 complex was purified as described in a previous study (Nguyen et al. 2020; and see Fig. 1C; Supplemental Fig. 1A). We found that Microprocessor (Fig. 1D) or cell extract-supplemented Microprocessor (Supplemental Fig. 1B) produced more pre-miRNA (F2) from pri-mir-146a_G than pri-mir-146a_C. This indicates that the G/C SNP directly reduced the production of pre-miRNA by Microprocessor. Interestingly, we found that the reduction in the amount of cleaved pre-mir-146a product was not correlated with an increase in the remaining pri-mir-146a_C substrate (Fig. 1D; Supplemental Fig. 1B,C). In fact, we observed that pri-mir-146a_C was degraded more than pri-mir-146a_G (Fig. 1D; Supplemental Fig. 1B,C). In addition, the patterns of the RNA products released from these two RNAs were quite different such that pri-mir-146a_G was cleaved at both its productive and unproductive sites, releasing pre-miRNA and unproductive products (UPC), respectively, whereas pri-mir-146a_C was mainly cleaved at its unproductive sites and thus generated more unproductive products (Fig. 1D,E; Supplemental Fig. 1B). The productive and unproductive products released from both pri-mir-146a_G and C were cloned and subjected to next-generation sequencing (NGS). The sites of productive and unproductive cleavages were then identified from NGS analysis (Supplemental Fig. 1D). We found that Microprocessor cleaved pri-mir-146a_G and C at the same productive sites, whereas it cleaved at different unproductive sites on the two substrates. In addition, time-course experiments showed that both pri-mir-146a_G and C were completely cleaved by Microprocessor after 2 h (Supplemental Fig. 1E,F), which suggests that both conformations were cleavable. These data indicate that Microprocessor can interact with pri-mir-146a_G at both the basal and apical junctions, whereas it interacts with pri-mir-146a_C mainly at the apical junction.

### The G/C SNP recruits DROSHA to the apical junction

**FIGURE 2.**
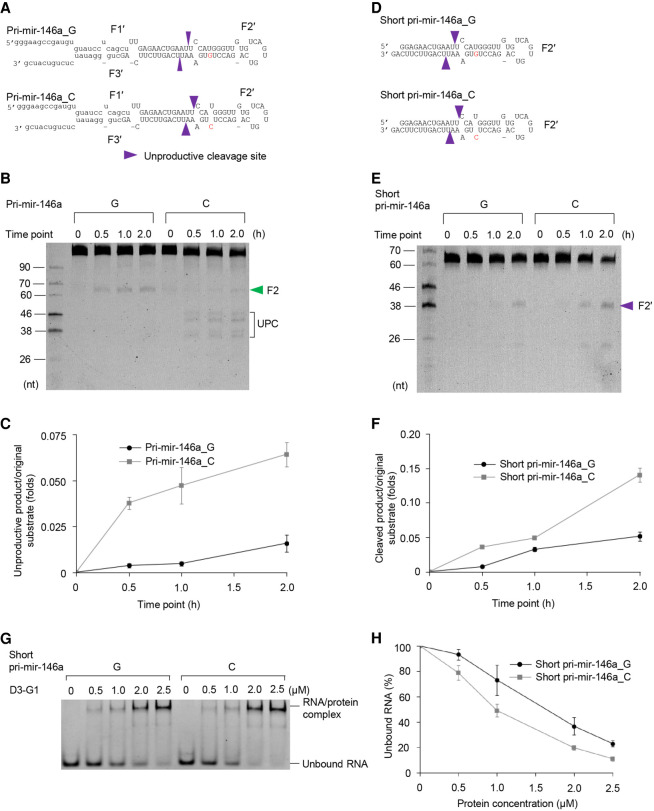
The G/C SNP recruits DROSHA to the apical junction. (*A*,*D*) The structures of pri-mir-146a_G and C (*A*), and short pri-mir-146a_G and C (*D*). The green and purple arrowheads indicate the productive and unproductive cleavage sites, respectively. (*B*,*E*) The cleavage of pri-mir-146a_G and C (*B*) and short pri-mir-146a_G and C (*E*) by D3–G1. Five pmol of each RNA was incubated with 15 pmol D3–G1 in 10 µL pri-miRNA processing buffer for 0.5, 1, and 2 h. UPC (in *B*) indicates the unproductive products. (*C*,*F*) The unproductive cleavage of D3–G1 (shown in *B* and *E*, respectively) was assessed in three independent experiments. (*G*) The EMSAs for D3–G1 and short pri-mir-146a_G and C. 1.5 pmol of each RNA was incubated with 5–25 pmol D3–G1 in 10 µL EMSA buffer. (*H*) Quantification of the EMSA data from *G*; the results were obtained from three independent experiments.

In order to understand how pri-mir-146a_C enhanced the apical orientation of Microprocessor, we purified the D3–G1 complex, which contained a fragment of DROSHA (i.e., 390–1365 amino acids) and a fragment of DGCR8 (i.e., 728–750 amino acids) (Fig. 1C; Supplemental Fig. 2). The G1 fragment helps to solubilize and stabilize the DROSHA fragment but does not contain any RNA-binding affinity (Nguyen et al. 2015). We then cleaved pri-mir-146a_G and C with the D3–G1 complex and found that it cleaved pri-mir-146a_C at the unproductive sites with a higher activity than pri-mir-146a_G (Fig. 2A–C). This suggests that DROSHA might be recruited to the apical junction of pri-mir-146a_C more efficiently.

To exclude any potential interference caused by the basal junction of pri-mir-146a on the unproductive cleavage, we synthesized two short pri-mir-146a substrates, which contained just the apical junction and upper stem (Fig. 2D). Similarly, we found that D3–G1 cleaved the short version of pri-mir-146a_C at the apical junction more efficiently than it cleaved the short version of pri-mir-146a_G (Fig. 2E,F). We then performed an electrophoretic mobility shift assay (EMSA) for the D3–G1 complex with these short versions of pri-mir-146a_G and C. We found that D3–G1 had a higher RNA-binding affinity to short pri-mir-146a_C when compared with short pri-mir-146a_G (Fig. 2G,H). This further supports our finding that pri-mir-146a_C facilitates the apical orientation of Microprocessor by recruiting DROSHA to the apical junction.

### The SNP G/C generates a noncanonical apical mGHG motif

**FIGURE 3.**
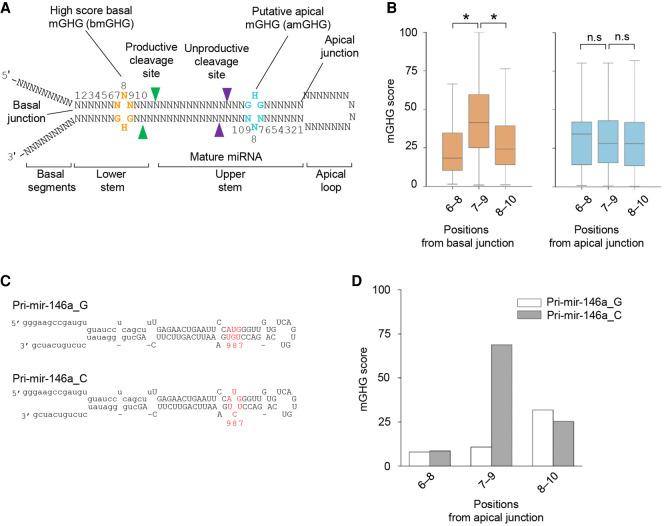
The SNP G/C generates a noncanonical apical mGHG motif in the apical region. (*A*) The positions of the canonical mGHG motifs at the basal junction in pri-miRNAs. The mGHG motif contains a mismatch at the eighth nucleotide from the basal junction. A putative apical mGHG motif might occur at the apical junction. (*B*) The mGHG scores were estimated for the mGHG motifs at the apical and basal junctions. Statistically significant and nonsignificant differences between the various data sets are indicated by an asterisk (*) and n.s., respectively (two-tailed *t-*test; mGHG scores of positions 6–8 vs. positions 7–9 from basal junction: *P* = 1.96 × 10^−50^, positions 7–9 vs. position 8–10 from basal junction: *P* = 3.83 × 10^−34^). (*C*) The putative apical mGHG motif in pri-mir-146a_C is shown in red. (*D*) mGHG scores of the mGHG motifs in positions 6 to 10 at the apical junction of pri-mir-146a_G or C.

It is known that mGHG (located between the basal junction and productive cleavage sites), interacts with the dsRBD of DROSHA, and thus enhances its productive cleavage (Fang and Bartel 2015; Kwon et al. 2019). This canonical mGHG motif was first described as a 3-bp motif, which contains a G–U or G–C, any unpaired nucleotide (except G), and a G–C pair at positions 7, 8, and 9 from the basal junction on the 3p-strand, respectively (Fig. 3A; Fang and Bartel 2015). Subsequently, the ability of an mGHG to enhance the productive cleavage of Microprocessor was scored using a range from 0 to 100 (Kwon et al. 2019). Here, we estimated mGHG scores for the trinucleotide motifs at both basal and apical junctions. Consistent with previous studies (Fang and Bartel 2015; Kwon et al. 2019), we showed that many human pri-miRNAs contain high score mGHG motifs in positions 7–9 from the basal junction (Fig. 3B). In contrast, the estimated mGHG scores for the trinucleotide motifs at the apical junction were relatively low (Fig. 3B). This is plausible because these low score apical mGHG motifs prevent DROSHA from locating and cleaving pri-miRNAs at the unproductive sites.

Here, we noted that the G/C SNP generated a mismatch at the eighth nucleotide from the apical junction (Fig. 3C; Supplemental Fig. 3). Thus, we hypothesized that the SNP might create a high mGHG score at the apical junction (Fig. 3C). Interestingly, we detected an unexpectedly strong mGHG motif (mGHG score = 68.8, UCU–GUA) in pri-mir-146a_C (Fig. 3D). This was in contrast to the relatively weak mGHG motif (mGHG score = 10.7, UGU–GUA) found in the same position in pri-mir-146a_G (Fig. 3D). We hypothesized that the noncanonical high score mGHG motif created by the G/C SNP (now called an apical mGHG motif), might efficiently recruit DROSHA to the apical junction of pri-mir-146a by interacting with its dsRBD.

### The apical mGHG motif recruits DROSHA to the apical region by interacting with its dsRBD

**FIGURE 4.**
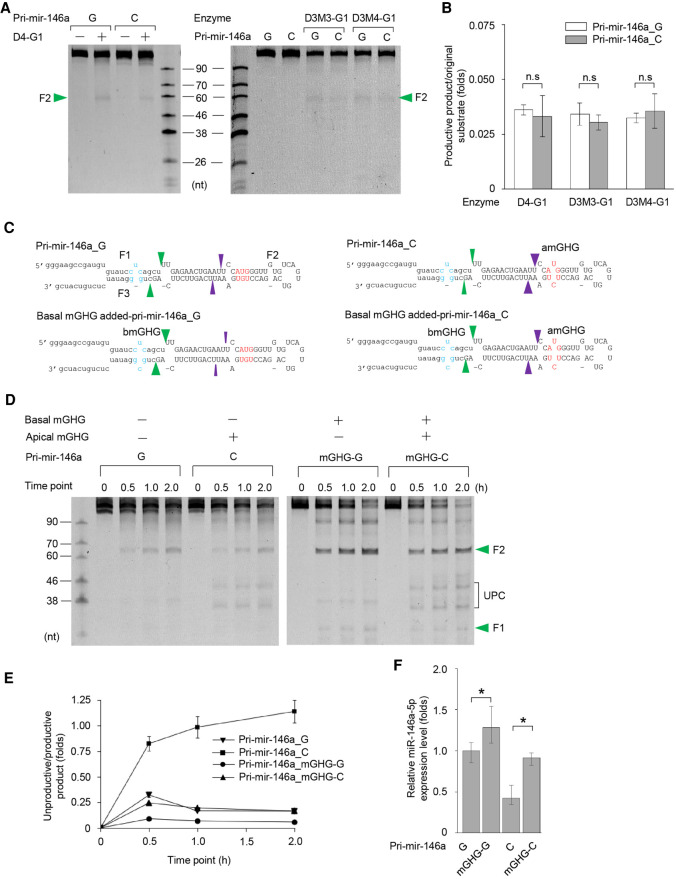
The apical mGHG motif recruits DROSHA to the apical junction via interaction with its dsRBD. (*A*) The cleavage of pri-mir-146a_G and C by mutant dsRBD-containing DROSHA. One pmol of each RNA was incubated with 6 pmol D3M3–G1, 6 pmol D3M4–G1, or 30 pmol D4–G1 in 10 µL pri-miRNA processing buffer. The green arrowheads indicate the productive cleavages. (*B*) The cleavage activity of the D3–G1 complexes (as shown in *A*) was calculated by estimating the ratio of the productive product to the original substrate from three independent experiments. No significant (n.s.) differences were found between the two data sets (two-tailed *t-*test). (*C*) The added basal mGHG motifs in pri-mir-146a_G and C are shown in blue. (*D*) Cleavage of the basal mGHG-added pri-mir-146a_G and C by Microprocessor (NLSD3–DGCR8). Five pmol of each RNA was incubated with 5 pmol Microprocessor in 10 µL pri-miRNA processing buffer for 0.5, 1, and 2 h. UPC indicates the unproductive products, whereas the green arrowheads indicate productive products. (*E*) The unproductive cleavage of Microprocessor (shown in *D*) was assessed from three independent experiments. (*F*) The qPCR-estimated miR-146a expression levels in human cells transfected with pcDNA3 plasmid expressing each of four pri-mir-146a in *D*. Statistically significant and nonsignificant differences between the various data sets are indicated by an asterisk (*) and n.s., respectively (two-tailed *t-*test; relative miR-146a expression level of pri-mir-146a_G vs. pri-mir-146a_mGHG-G: *P* = 0.043, pri-mir-146a_C vs. pri-mir-146a_mGHG-C: *P* = 0.029).

We generated a dsRBD-deleted DROSHA fragment (D4, amino acids 390–1260) and purified a D4–G1 complex (Fig. 1C; Supplemental Fig. 4A). Unlike D3–G1, the D4–G1 complex no longer cleaved pri-mir-146a_G and C at the unproductive sites (Fig. 4A). Instead, it cleaved these substrates at the productive sites with a similar activity (Fig. 4A,B). In addition, we found that D4–G1 cleaved the short versions of pri-mir-146a_G and C similarly (Supplemental Fig. 4B,C). These data support the hypothesis that the apical mGHG–dsRBD interaction is the main driving force causing DROSHA to reside at the apical junction in pri-mir-146a_C, which results in the unproductive cleavages.

We then purified two other mutant D3–G1 complexes, D3M3–G1, and D3M4–G1, which contained point mutations in the dsRBD (Fig. 1C; Supplemental Fig. 4A). These amino acids are essential for the mGHG–dsRBD interaction (Kwon et al. 2019). Like D4–G1, the D3M3–G1, and D3M4–G1 complexes also cleaved pri-mir-146a_G and C similarly at the basal junction (Fig. 4A,B). These observations once again demonstrate that the apical mGHG motif facilitated cleavage by Microprocessor at the apical junction via interacting with its dsRBD.

We then utilized a more potent mGHG motif (CUC–GCG; mGHG score = 95.9) (Fang and Bartel 2015; Kwon et al. 2019) at the basal junction of the pri-mir-146a_G and C substrates (Fig. 4C), and demonstrated that Microprocessor cleaved these substrates at the productive sites more efficiently (Fig. 4C–E). These data again indicate that the mGHG motifs are an important factor that alters the orientation of DROSHA on pri-mir-146a, and hence that drives Microprocessor to cleave at either the basal or apical junction.

Finally, we expressed the four pri-mir-146a variants shown in Figure 4C, in HCT116 cells. This was achieved by transfecting pCDNA3 plasmids containing DNA from the respective region of each pri-mir-146a variant. We showed that the level of miRNA expression from each pri-mir-146a variant (estimated by qPCR), correlated with the levels of the pre-mir-146a variants determined by the *in vitro* processing assays (Fig. 4F). In addition, the RNA levels of the four pri-mir-146a variants (estimated from the transfected cells), were somewhat similar and their levels correlated with the remaining pri-miRNA substrates in the in vitro processing assays (Supplemental Fig. 4D). These data support the hypothesis that the difference in miRNA expression from the four pri-mir-146a variants is due mainly to the difference in the productive and unproductive cleavages.

## DISCUSSION

**FIGURE 5.**
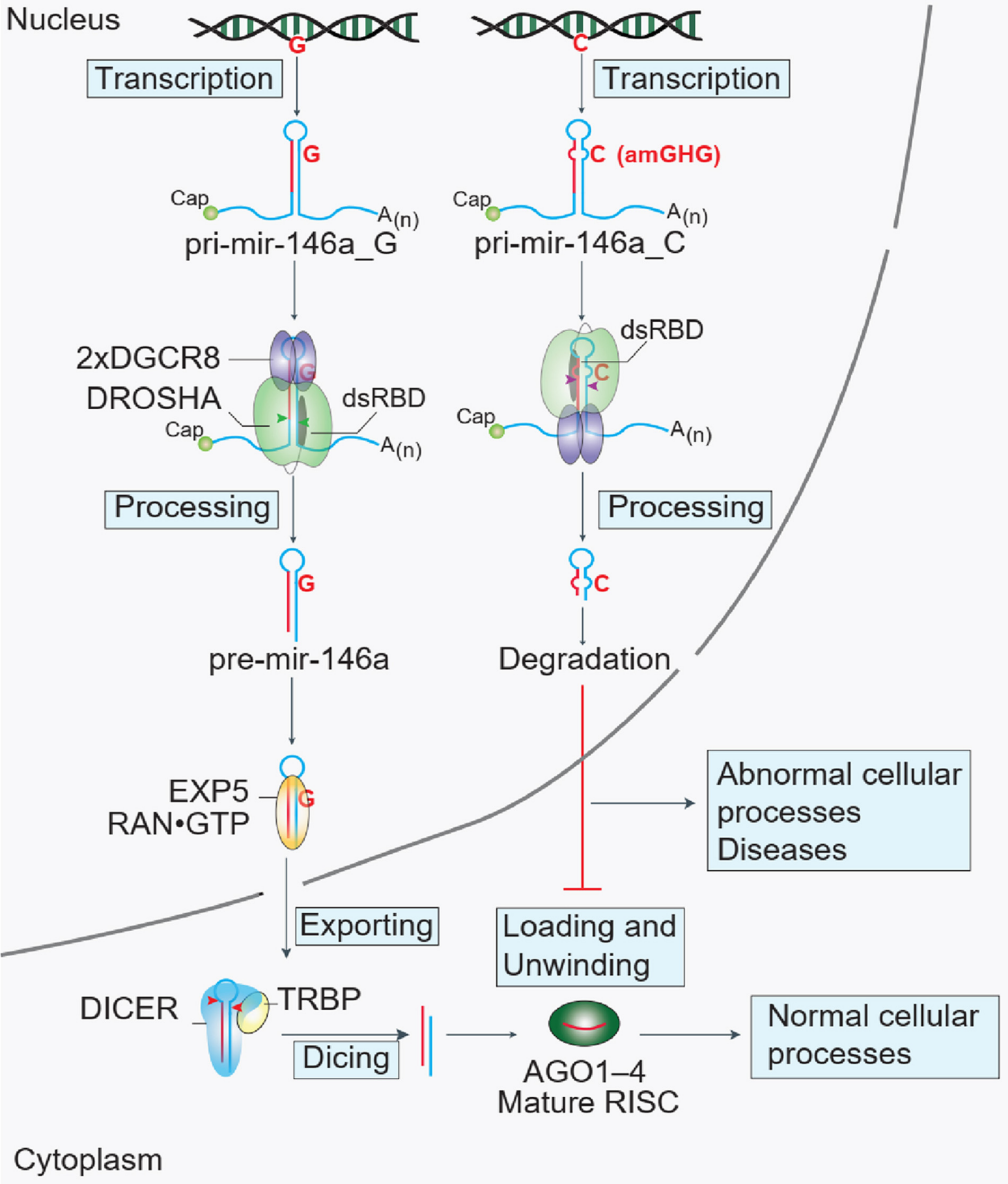
Schematic representation of a hypothetical model to explain how the G/C SNP (rs2910164) reduces the expression of miR-146a. The G and C alleles located in the *5q33* locus of chromosome 5 are different in one nucleotide. These two alleles are transcribed into two different pri-mir-146a molecules. Pri-mir-146a_C has a strong amGHG motif, which interacts with the dsRBD of DROSHA, and in this way, it efficiently recruits DROSHA to the apical junction. Therefore, the Microprocessor cleaves pri-mir-146a_C more at the unproductive sites, generating a short hairpin, which is an unproductive product. This unproductive product does not produce miR-146a, and it might be degraded in human cells. As pri-mir-146a_G is cleaved more at the productive sites, it generates more pre-mir-146a, which is in turn passed through the DICER and Ago steps to become mature miR-146a. RISC: RNA-induced silencing complex.

Since pri-miRNAs are rather symmetrical and contain both basal and apical junctions, multiple mechanisms ensure that DROSHA in the Microprocessor complex is placed at the basal junction. In this orientation, DROSHA cleaves pri-miRNAs correctly at their productive sites to produce pre-miRNAs. In human pri-miRNAs, the mGHG motif is highly enriched in positions 7–9 of the lower stem and it is conserved across many organisms (Fang and Bartel 2015). This motif is one of the critical RNA elements that help DROSHA to bind to the basal junction, and it is therefore called the basal mGHG motif, bmGHG. In our study, we showed that high score mGHG motifs appear only rarely at the apical junction of human pri-miRNAs, and so DROSHA is prevented from being located at the apical junction (Fig. 3B). We also identified an unexpected mGHG motif (formed by the SNP, rs2910164), at the apical junction of pri-mir-146a. This so-called apical mGHG, amGHG, recruits DROSHA to the apical junction by interacting with its dsRBD. Unlike the bmGHG motif, which facilitates the productive cleavage, the amGHG motif enhances the unproductive cleavage. This SNP-derived amGHG motif helps to explain the molecular mechanism involved in the down-regulation of miR-146a in various human diseases (Fig. 5).

Multiple RNA elements (including UG, UGU, mGHG, seedMW, midMW, CNNC, and AIL), are known to affect the cleavage state of Microprocessor via either DROSHA or DGCR8 (Auyeung et al. 2013; Fang and Bartel 2015; Nguyen et al. 2015, 2018, 2019, 2020; Kim et al. 2018; Kwon et al. 2019; Dang et al. 2020; Li et al. 2020). We have previously demonstrated that the action of an RNA element is dependent on its position. Indeed, by placing a motif at either the basal or apical junction we could alter the orientation of Microprocessor. For example, by switching the basal UG to an apical UG, or the apical UGU to a basal UGU, the orientation of Microprocessor could be manipulated (Nguyen et al. 2015). In this new study, we demonstrated that the amGHG motif generated by the G/C SNP, rs2910164, in pri-mir-146a could also alter the orientation of Microprocessor. Being able to identify and investigate RNA modifications, SNPs, or mutations that might result in any of the RNA motifs being located in wrong positions, which subsequently influences miRNA biogenesis, is crucial for exploring the molecular mechanisms involved in the changes in cellular function observed in human diseases.

## MATERIALS AND METHODS

### The mGHG scores at the basal and apical junctions

Human pri-miRNA sequences were obtained from MirGeneDB v2.0 (Fromm et al. 2020). The RNAs were folded using RNAfold (using the default parameters) (Lorenz et al. 2011). The apical junction (or basal junction) of the pri-miRNAs was determined as being the ssRNA/dsRNA junction between the loop (or the basal segments) and the stem of the pri-miRNAs. Trinucleotide windows at positions 6–8, 7–9, and 8–10 on each strand of the pri-miRNA stem were collected from the apical or basal junction. The mGHG scores were then assigned for each of these trinucleotide motifs, using estimated mGHG scores from a previous report (Kwon et al. 2019). However, trinucleotide motifs that contained bulges were not assigned mGHG scores.

### Recombinant protein preparation

Purified proteins were prepared as described in our previous study (Nguyen et al. 2020). We cotransfected HEK293E cells with a mixture of pXab–D3 and pXC–G1 or pXab–NLSD3 and pXG–DGCR8 for expressing the D3–G1 or NLSD3–DGCR8 complexes, respectively. These two complexes were purified using the Ni-NTA resin and Bio-Rad UNOsphere Q beads. The D3–G1 complex was further purified using a Superdex 200 Increase 10/300 GL gel filtration column, as described previously (Nguyen et al. 2020). We mutated pXab–D3 to obtain pXab-D4 (390–1260 amino acids), pXab–D3M3, and pXab–D3M4. The point mutations in D3M3 and D3M4 were QQ1266–67AI/CL1269–70AR/R1273S/EPDI1278–81GGSS and QQ1266–67LE/L1270H /R1273S/EPDI1278–81GGSH, respectively. We purified the D4–G1, D3M3–G1, and D3M4–G1 complexes, as described for D3–G1.

### Preparation of the RNA substrates

We conducted in vitro transcription (IVT) reactions to synthesize RNA substrates from the T7 promoter-containing dsDNAs using T7 RNA polymerase. The NTP and T7 RNA polymerases were from the MEGAscript T7 Kit (Invitrogen, AMB13345). The T7-containing dsDNAs were generated by PCR using the primers and DNA templates shown in Supplemental Table 1. Each IVT reaction mixture (20 µL) contained 200 ng dsDNA, 10 mM of each NTP, and 2 µL stock T7 RNA polymerases. The IVT reactions were incubated at 37°C for 12–16 h, after which the resulting RNAs were separated via 10% urea-PAGE. The RNA bands were then gel eluted and purified with isopropanol. The purified RNAs were quantified using a Nanodrop spectrophotometer, and their integrity was further evaluated via 10% urea-PAGE. Finally, the purified RNAs were stored at −80°C until required.

### Pri-miRNA processing assay

The pri-miRNA was mixed with Microprocessor in 10 µL of a 50 mM Tris-HCl (pH 7.5) buffer supplemented with 150 mM NaCl, 10% glycerol, 0.2 µg/µL BSA, 2 mM MgCl_2_, and 1 mM DTT. The amounts of RNAs and enzymes used are indicated in the figures or figure legends. The reactions were carried out at 37°C for 2 h, after which the reaction mixtures were treated with 10 µL 2× TBE-urea buffer and immediately kept on ice. Subsequently, the urea-treated reaction mixtures were added to 20 µg proteinase K and the resulting mixtures were incubated at 37°C for 15 min, 50°C for 15 min, 95°C for 5 min, and finally on ice. The reaction mixtures were finally loaded onto a prerun 12% urea-PAGE in 1× TBE buffer. The urea-PAGE was stained with SYBR Green II RNA Gel stain (Invitrogen, S7564) for 5 min, after which images were acquired using the Bio-Rad Gel Doc XR+ system and the intensity of RNA bands was measured using Image Lab (Bio-Rad) software 6.0.

### Electrophoretic mobility shift assay (EMSA)

Each RNA substrate (1.5 pmol) and different amounts of enzyme were mixed in a 10 µL reaction solution containing 50 mM Tris-HCl (pH 7.5), 150 mM NaCl, 0.2 mg/ml BSA, 10% glycerol, 2 mM EDTA, and 1 mM DTT. The mixture was incubated on ice for 60 min, after which 5 µL of the reaction mixture was loaded onto a 4% native PAGE gel, which was run at 4°C using a precooled 1× TBE buffer at 8 V/cm for 45 min. After running, the gel was stained with ethidium bromide. Images were acquired and RNA band intensities were measured, as described above.

### Plasmid construction, transfection, and quantitative-PCR (qPCR)

The DNA sequences encoding the pri-mir-146a variants (see Supplemental Table 1) were cloned into the pcDNA3 plasmid. The constructed pri-mir-146a plasmids were confirmed by Sanger sequencing. Each plasmid (2 µg) was cotransfected with 0.5 µg pcDNA3-pri-mir-1226 as an internal control into HCT116 cells in a 60-mm dish using 7.5 µl Lipofectamine 3000 reagent (Thermo Scientific, L3000015). The transfected cells were harvested 48 h after transfection and total RNA was extracted using TRIzol (Ambion, 15596018).

Total RNA (50 ng) was used in a reverse-transcription reaction using a stem–loop primer designed for each miRNA sequence as described in a previous study (Chen et al. 2005). qPCR was conducted using the iTaq Universal SYBR Green Supermix (Bio-Rad). RT and qPCR primer sequences are shown in Supplemental Table 2.

### RNA cloning and bioinformatics analysis

The productive and unproductive products of the Microprocessor-cleavage assays (Fig. 1D) were gel-eluted and purified using isopropanol. The purified RNAs were then ligated to the 3′-adapter, 4N-RA3 (/5rApp/NN NNTGGAATTCTCGGGTGCCAAGG/3ddC/) using T4 RNA ligase 2, truncated KQ (NEB, M0373L). The 4N-RA3-ligated RNAs were reverse-transcribed (RT) using SuperScript IV Reverse Transcriptase (Invitrogen, 18090050) and 6N-cirRTP (/5Phos/NNNNNNGATCGTCGGACTGTAGAACTCTGAAC/iSp18/CCTTGGCACCCGAGAATTCCA) primer. Subsequently, 1 mM NaOH was added to each RT reaction mixture and it was incubated at 95° for 10 min to remove the RNAs. The NaOH-treated mixtures were then separated via urea-PAGE, after which the cDNAs were cut from gel and purified using isopropanol. The purified cDNAs were then circularized using CircLigase ssDNA Ligase (Epicentre, CL4115K) and amplified using the primer pairs of the RP1 and RPI primers. Finally, the resulting DNA products were sequenced using next-generation sequencing (Illumina NextSeq 500).

The 3′- and 5′-adapters were first removed from the raw reads using cutadapt (cutadapt -a TGGAATTCTCGGGTGCCA AGG -AGATCGTCGGACTGTAGAACTCTGAAC) (Martin 2011). Fastq-join (default parameters) was used to join the trimmed paired-end reads. The reads with low quality were discarded using fastq_quality_filter (-q 30 -p 90), and any duplication of the reads was removed using fastx_collapser (default parameters) (FASTX-Toolkit, http://hannonlab.cshl.edu/fastx_toolkit/index.html, version 0.0.13). The barcode sequences at both ends of the reads were then trimmed using cutadapt, and the trimmed reads were mapped to the sequences of pri-mir-146a_G and C using BWA (Li and Durbin 2009). Only perfectly aligned reads were collected for further analysis (Supplemental Table 3).

### Statistics and reproducibility

Statistical tests were performed for experiments conducted in triplicate. Quantitative data were expressed as mean ± SD and *P*-values were estimated with the two-tailed *t*-test.

## SUPPLEMENTAL MATERIAL

Supplemental material is available for this article.

## Supplementary Material

Supplemental Material
