# Supplementary material for: Human disease-associated single nucleotide polymorphism changes the orientation of DROSHA on pri-mir-146a
Source: RNA. 2020 Dec;26(12):1777–86. doi: 10.1261/rna.077487.120 (PMC7668254; doi:10.1261/rna.077487.120)
Supplement: Supplemental Material [file supp_26_12_1777__index.html]

Human disease-associated single nucleotide polymorphism changes the orientation of DROSHA on pri-mir-146a — Supplemental Material 

# Human disease-associated single nucleotide polymorphism changes the orientation of DROSHA on pri-mir-146a

## Supplemental Material

- Supplemental\_Figures\_.pdf
- Supplemental\_Table\_1.xlsx
- Supplemental\_Table\_2.xlsx
- Supplemental\_Table\_3.xlsx
