# Supplementary material for: Human disease-associated single nucleotide polymorphism changes the orientation of DROSHA on pri-mir-146a
Source: RNA. 2020 Dec;26(12):1777–86. doi: 10.1261/rna.077487.120 (PMC7668254; doi:10.1261/rna.077487.120)
Supplement: Supplemental Material [file supp_077487.120_Supplemental_Figures_.pdf]

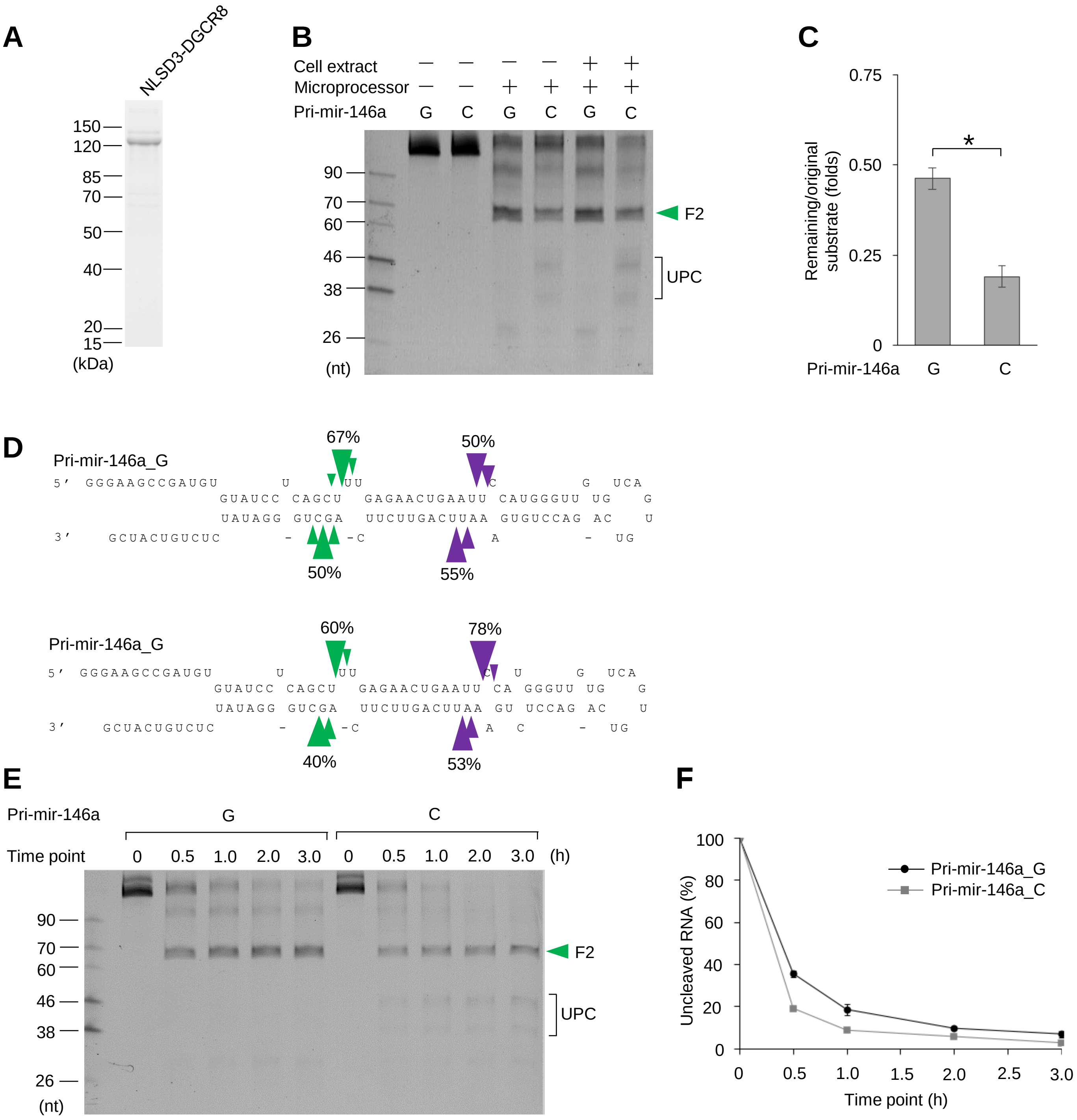

Supplemental Figure 1. The purified Microprocessor and its pri-mir-146a processing

(A) Purified NLSD3-DGCR8 complex on SDS-PAGE.

(B) The cleavage of pri-mir-146a_G and C by Microprocessor with/without cell extract supplemented. The purified Microprocessor was first bound with the IgG sepharose beads. Subsequently, the HEK293E cell extract was added to the Microprocessor-bound beads. After being washed with the washing buffer containing 150 mM NaCl, the cell extract-supplemented Microprocessor was assayed with pri-mir-146a_G or C on the beads.

(C) The ratio of remaining to original pri-mir-146a_G and C in the processing assays with NLSD3-DGCR8 was estimated from three independent experiments. Statistically significant and nonsignificant differences between two data sets were indicated by the asterisk (*) and (n.s), respectively (two-tailed *t*-test, $p = 0.00042$).

(D) Analysis of Microprocessor cleavage sites on pri-mir-146a_G and C. The green and purple arrowheads indicate the productive and unproductive cleavages, respectively.

(E) Time-course cleavage assays for Microprocessor and pri-mir-146a_G or C. 1.5 pmol of each substrate was incubated with 3 pmol of Microprocessor in 10 μL pri-miRNA processing buffer for 0.5, 1, 2, and 3 h. UPC indicates unproductive products.

(F) Quantification of cleavage data from (E). The results were obtained from three independent experiments.

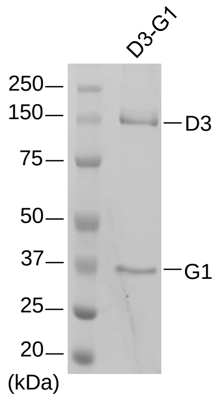

Supplemental Figure 2. Purified D3-G1 complex on SDS-PAGE.

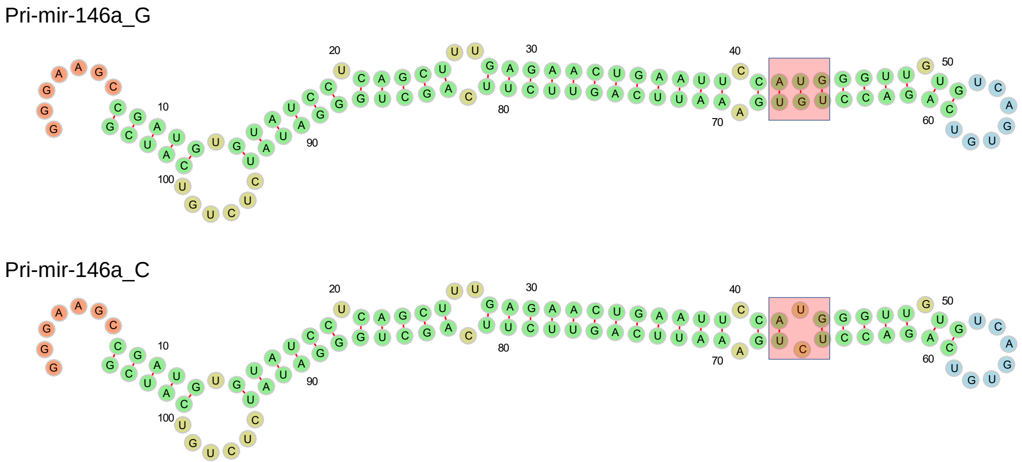

Supplemental Figure 3. Folded structure of pri-mir-146a_G and C obtained using RNAfold (Lorenz et al., 2011).

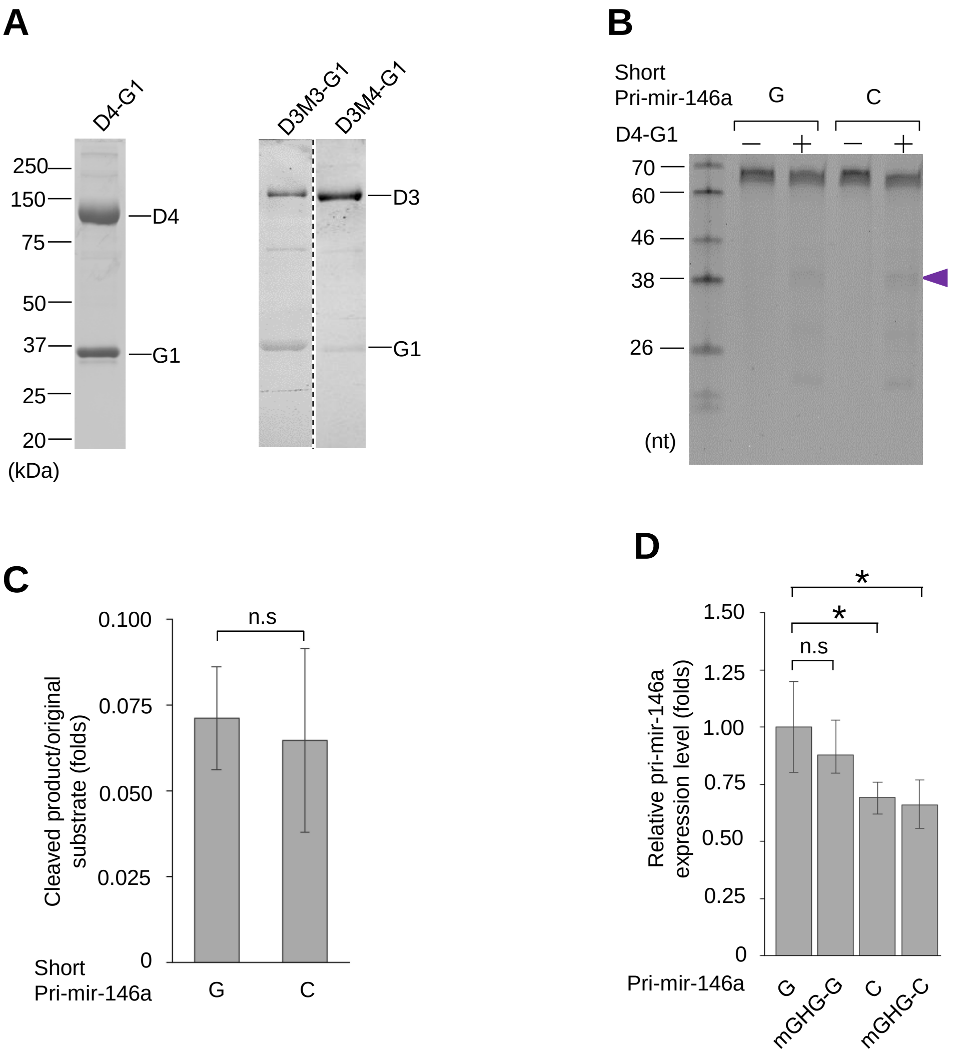

Supplemental Figure 4. The purified mutant D3-G1 complexes and their pri-mir-146a processing

(A) Purified D4-G1, D3M3-G1 and D3M4-G1 complexes on SDS-PAGE.

(B) The cleavage of short pri-mir-146a_G and C by the D4-G1 complex, respectively. One pmol of each substrate was incubated with 30 pmol of D4-G1 in 10 μL processing buffer.

(C) The cleavage efficiency of the D4-G1 complex in (B) was accessed from three independent experiments. Statistically significant and nonsignificant differences between two data sets were indicated by the asterisk (*) and (n.s), respectively (two-tailed *t*-test).

(D) The qPCR-estimated pri-mir-146a levels from the experiments described in Figure 4C. Statistically significant and nonsignificant differences between the various data sets were indicated by an asterisk (*) and n.s., respectively (two-tailed *t*-test; relative pri-mir-146a expression level of pcDNA3-pri-mir-146a_G vs. pcDNA3-pri-mir-146a_C: $p = 0.017$; pcDNA3-pri-mir-146a_G vs. pcDNA3-pri-mir-146a_mGHG-G: $p = 0.032$).
